# Association between physical exercise and cognitive frailty among community-dwelling older adults: the mediating role of self-efficacy

**DOI:** 10.3389/fpubh.2026.1903378

**Published:** 2026-08-06

**Authors:** DanDan Liang, Ruiying Jia, Zhen Wu, Tingting Fan, Yanhong Pan

**Affiliations:** 1 Nursing Department, Second Affiliated Hospital, School of Medicine, Zhejiang University, Hangzhou, China; 2 Xinyang Vocational and Technical College, School of Nursing, Xinyang, China

**Keywords:** cognitive frailty, community-dwelling older adults, mediation effect, physical exercise, self-efficacy

## Abstract

**Purpose:**

This study aimed to examine the relationship among physical exercise, self-efficacy, and cognitive frailty, and to test the mediating role of self-efficacy in the association between physical exercise and cognitive frailty among community-dwelling older adults.

**Methods:**

This cross-sectional survey employed a convenience sampling method to recruit community-dwelling older adults from nine communities in Henan Province, China. Data were collected from January to May 2026. The measurements included a general information questionnaire, the Fried Phenotype (FP), the Montreal Cognitive Assessment (MoCA), the Physical Activity Rating Scale-3 (PARS-3), and the General Self-Efficacy Scale (GSES). SPSS 27.0 and R software were used for statistical analysis.

**Result:**

A total of 570 questionnaires were distributed, and after excluding invalid responses, 563 valid questionnaires were retained. The prevalence of cognitive frailty among community-dwelling older adults was 21.3%. Univariate analysis revealed that age, marital status, education level, smoking, drinking, average monthly income, sleep quality, chronic diseases, subjective cognitive decline (SCD), physical exercise score, and self-efficacy score were significantly associated with cognitive frailty (*p* < 0.05). Mediation analysis further showed that self-efficacy significantly mediated the association between physical exercise and cognitive frailty, with the mediation effect accounting for 35.17% of the total effect.

**Conclusion:**

The prevalence of cognitive frailty among community-dwelling older adults was relatively high. Physical exercise was directly associated with cognitive frailty and also indirectly associated with it through self-efficacy, with the indirect effect accounting for 35.17% of the total effect.

## 1 Introduction

With improvements in socioeconomic and healthcare conditions, along with extended life expectancy, population aging has become a major global concern. The global population aged 60 years and older is projected to increase from 1 billion in 2020 to 1.4 billion by 2030, and to 2.1 billion by 2050, with this growth occurring at an unprecedented rate, particularly in developing countries (1). Since China became an aging society in 1999, the population aged 60 years and older had reached 264 million by the end of 2020. This number is projected to grow to 400 million by 2032 and exceed 500 million by 2048 (2). As the older adult population continues to grow, the World Health Organization introduced the concept of “healthy aging” in 1990 in response to the challenges of population aging. In 2002, this concept evolved into “active aging,” with the aim of fully realizing the potential of older adults’ healthy life expectancy. With increasing research on population aging, it is now recognized as both an opportunity and a challenge. Extended life expectancy not only creates opportunities for older adults and their families but also contributes positively to society—for example, through continued education, new career opportunities, and an expanded labor force (3). However, the opportunities presented by population aging depend largely on maintaining good health. When extended longevity is accompanied by poor health or high care dependency, the negative impact on both older adults and society grows substantially, which can become a major obstacle to achieving active aging (4).

### 1.1 Background

Cognitive frailty is a heterogeneous clinical manifestation characterized by the simultaneous presence of physical frailty and cognitive impairment (defined as a Clinical Dementia Rating score of 0.5), with the exclusion of Alzheimer‘s disease or other types of dementia. It has emerged as a new target for healthy aging (5). Frailty is a critical transitional state in the dynamic process from robustness to aging-related functional decline. During this process, the decline and dysfunction of physiological reserves in older adults lead to increased vulnerability and susceptibility to diseases, as well as weakened stress resistance and reduced homeostatic capacity. These changes make it increasingly difficult to sustain and repair the aging body (6). Cognitive impairment refers to the decline of cognitive functions—including thinking, memory, reasoning, and planning—caused by various etiological factors (7). Early cognitive impairment mainly includes mild cognitive impairment (MCI) and subjective cognitive decline (SCD). MCI is characterized by cognitive decline without significant functional impairment and does not interfere with activities of daily living (7, 8); SCD refers to self-perceived cognitive decline that does not meet the criteria for MCI or dementia on objective assessments (9). Studies have shown that physical frailty and cognitive impairment often occur concurrently in the same older adult and interact with each other, eventually forming a vicious cycle (10, 11). Based on this, the International Expert Consensus Group (the International Institute of Nutrition and Aging and the International Association of Gerontology and Geriatrics) proposed the concept of cognitive frailty in 2013. Cognitive frailty includes two subtypes: reversible and potentially reversible cognitive frailty. Reversible cognitive frailty is characterized by SCD and/or biomarker positivity, whereas potentially reversible cognitive frailty involves MCI (12). The factors associated with cognitive frailty in older adults are complex, mainly including vascular risk factors, nutritional disorders, environmental factors, genetics, hormonal and metabolic factors, anxiety, depression, and unhealthy lifestyle habits, among others (13–15). Moreover, cognitive frailty in older adults is a risk state for multiple adverse health outcomes, including dementia, disability, falls, and even death (16, 17). Therefore, it is necessary to actively identify effective strategies to prevent physical frailty and improve cognitive function.

In many community-based comprehensive care programs for older adults, physical exercise is commonly incorporated as a key component to address aging-related frailty or functional decline (18–20). Physical exercise refers to physical activity of a certain intensity, frequency, and duration designed to improve physical health. Participation in physical exercise often induces feelings of excitement and pleasure, which may help alleviate negative emotional states (21, 22). Studies have shown that physical exercise offers multiple benefits for older adults, including improving physical flexibility, enhancing daily functioning, improving gait, reducing falls, and increasing bone density, thereby alleviating frailty (23, 24). In addition, long-term physical exercise can help maintain and improve cognitive function in older adults, delay cognitive aging and neurodegeneration, and is crucial for preserving executive function (25, 26). Other studies have shown that physical exercise improves neurobiological factors associated with cognitive frailty—such as glucose metabolism, sarcopenia, and insulin resistance, among others—which may help delay the onset and progression of cognitive frailty (27–29). Therefore, physical exercise may be an effective strategy to prevent or delay cognitive frailty in older adults.

Self-efficacy is a core concept of social cognitive theory, referring to individuals’ confidence in their ability to mobilize the skills needed to accomplish specific tasks (30). According to the triadic reciprocal determinism framework, cognition, behavior, and environment influence each other (31). Thus, physical exercise—as both a behavior and a social environmental factor—and self-efficacy can influence each other bidirectionally. During physical exercise, older adults experience improvements in physical function and cognitive ability, which may enhance their sense of value and control within their family or community roles. Moreover, regular exercise itself fosters feelings of perseverance and a sense of achievement, which can strengthen their confidence in coping with stressful events and improve their self-efficacy (32). Higher self-efficacy can improve health self-management in older adults and facilitate adherence to health-promoting behaviors, thereby promoting physical and mental health and reducing the likelihood of frailty (33). In addition, the brain reserve hypothesis (34) suggests that various factors—including social networks, education, leisure activities, and exercise—contribute to cognitive reserve and help maintain cognitive function. These factors are positively influenced by higher self-efficacy, which itself may serve as a protective factor for brain reserve, enabling individuals to cope with disease-related neuropathological changes without progressing to MCI or dementia (35). In summary, physical exercise may be directly associated with cognitive frailty in older adults and may also be indirectly associated with it through self-efficacy.

In the context of population aging, cognitive frailty among community-dwelling older adults poses a significant challenge to achieving active aging. Given its potentially reversible nature, early assessment and intervention are warranted. This study aimed to examine the relationship among physical exercise, self-efficacy, and cognitive frailty in community-dwelling older adults, and to explore the mediating role of self-efficacy in the association between physical exercise and cognitive frailty. The findings may provide a theoretical basis for community healthcare providers to develop strategies for delaying cognitive frailty, thereby supporting the broader goal of healthy aging.

### 1.2 Theoretical basis

**Figure 1:**
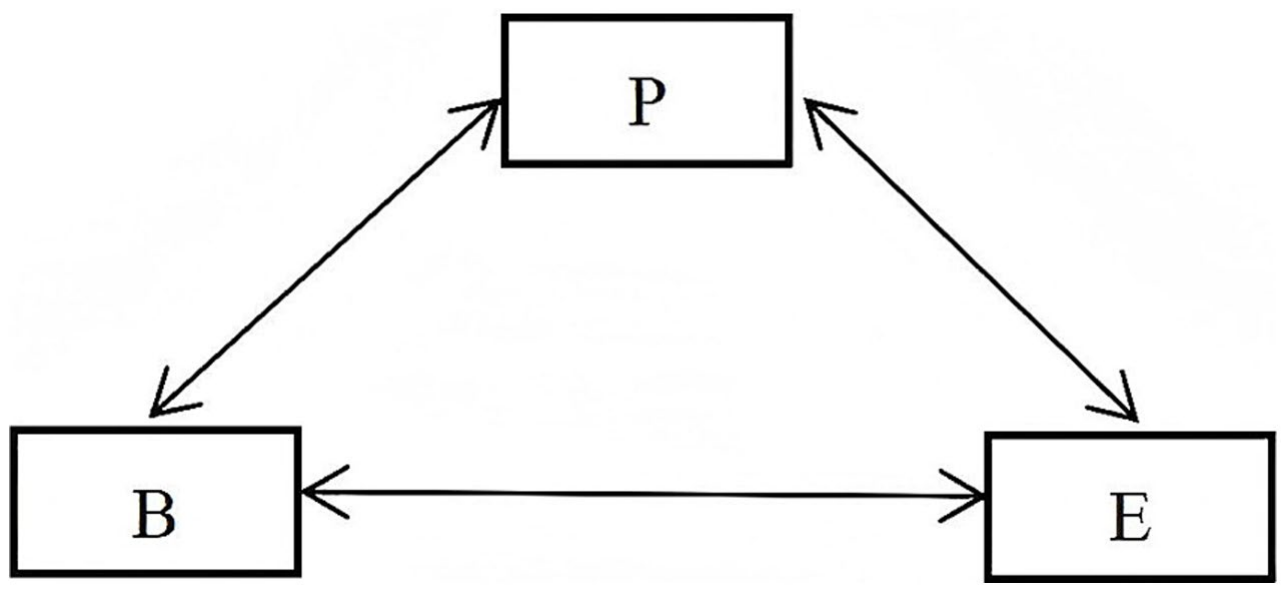
The triadic reciprocal determinism mode. P, People; B, Behavior; E, Environment.

The triadic reciprocal determinism was proposed by Bandura (31). This framework posits that environment, personal factors (including cognition, motivation, physiology, emotion, etc.), and behavior are relatively independent, yet they continuously interact with and mutually determine each other. The triadic reciprocal determinism mode is shown in Figure 1. Personal factors exert a strong influence on behavior. For instance, if individuals perceive a low probability of success in a given situation, they are unlikely to take action, thereby reducing their actual chances of success. Similarly, personal factors and the environment influence and shape each other. When individuals believe they can succeed in a particular environment, they are more likely to choose or even actively create such an environment. Behavior serves as the mediator between individuals and their environment, acting as the means through which individuals select, modify, or create environments. At the same time, behavior is also constrained by environmental conditions. The specific manifestations and mechanisms of these interactions vary across different situations, individuals, and activities (36, 37).

Drawing on the triadic reciprocal determinism framework, this study posits that self-efficacy and the contextual stressors associated with cognitive frailty jointly influence individuals’ subjective initiative, which in turn shapes their engagement in physical exercise. Physical exercise may enhance self-efficacy by improving emotional states and physiological arousal in older adults, and may also transform the cognitive frailty context by altering cognitive appraisals. Based on this rationale, the following hypothesis is proposed:

*Hypothesis* 1: Physical exercise is negatively correlated with cognitive frailty among community-dwelling older adults.

*Hypothesis* 2: Physical exercise is positively correlated with self-efficacy among community-dwelling older adults.

*Hypothesis* 3: Self-efficacy is negatively correlated with cognitive frailty among community-dwelling older adults.

*Hypothesis* 4: Self-efficacy significantly mediates the association between physical exercise and cognitive frailty among community-dwelling older adults.

## 2 Materials and methods

### 2.1 Designs and participants

This cross-sectional study employed a convenience sampling method. A total of nine communities in Kaifeng City, Henan Province, China, were selected as the survey sites. Data collection was conducted from January to May 2026.

The selection criteria for these communities were: (1) accessibility to the research team within a 20 km radius of the study coordination center; (2) community population size of at least 2,500 residents aged 60 years or above, to ensure sufficient recruitment capacity; and (3) willingness of local community health service centers to collaborate and provide logistical support for the survey. According to the China National Bureau of Statistics urban–rural classification, seven of the selected communities were urban and two were rural.

Inclusion criteria: (1) age ≥ 60 years; (2) residence in the target community for at least 1 year; (3) ability to walk independently without walking aids (e.g., cane, walker) and to complete the walking test required in this study; and (4) provision of written informed consent and voluntary participation.

Exclusion criteria: (1) severe hearing impairment (defined as inability to understand conversational speech at normal volume even with hearing aids) or severe visual impairment (defined as inability to read printed text at a distance of 40 cm even with corrective lenses), which would prevent completion of the survey; (2) severe communication impairment or diagnosed dementia that precluded cooperation; and (3) documented diagnosis of advanced cancer (Stage IV or terminal stage) or end-stage organ failure (e.g., New York Heart Association Class IV heart failure, end-stage renal disease requiring dialysis, or severe chronic obstructive pulmonary disease with oxygen dependence), or obvious physical defects that would interfere with study participation.

The sample size is calculated as follows:

$$N=\frac{{u_{a/2}^{2}}^{*}\pi (1-\pi )}{\delta^{2}}$$

Where *a*/2 is the bilateral *u* value of *α* at the specified inspection level. In this study, *α* = 0.05, *u*_*a*/2_ = 1.96. *π* is the estimated value of the expected probability. Based on previous data, the prevalence of cognitive frailty among community-dwelling older adults assessed by FP and MoCA in China was 9.6% (38), then *π* = 0.096. *δ* is an allowable error, this study takes *δ* = 3%. The sample size was calculated as *N* = 370. Accounting for a 20% attrition rate, the final target sample size was set at 444 participants.

### 2.2 Data collection

All investigators received standardized training conducted by the research team, including detailed instructions on the survey purpose, content, and completion procedures. Questionnaires were administered on-site using a paper-and-pencil format. A total of 570 questionnaires were distributed, all of which were returned, yielding 563 valid responses after data cleaning. This exceeded the pre-calculated target of 444 and represented an effective response rate of 98.77%. The additional questionnaires were distributed to account for potential data quality issues and to further enhance statistical power.

### 2.3 Measurements

Data was collected using the general information questionnaire, FP, MoCA, PARS-3 and GSES. Except for the general information questionnaire, the other scales were mature scales at home and abroad.

#### 2.3.1 The general information questionnaire

The general information questionnaire was developed by the research team based on a literature review and the study objectives. It covered 11 items: gender, age, marital status, education level, smoking, drinking, residence form, average monthly personal income, sleep quality, chronic diseases, and SCD. SCD was assessed using a single self-report item: “Do you perceive a decline in your memory compared with one year ago?”.

#### 2.3.2 Assessment of cognitive frailty

The FP was developed by Fried et al. (6) and comprises five components: unintentional weight loss, slow walking speed, low grip strength, self-reported exhaustion, and low physical activity. Each component is scored 1 if present and 0 otherwise, yielding a total score ranging from 0 to 5. A score of 0 indicates no frailty.

MoCA was developed by Nasreddine et al. (39). The Chinese version, translated and adapted by Wang Wei and colleagues, is the most widely used version in China (40). The MoCA assesses seven cognitive domains: visuospatial and executive function, naming, attention, language, abstraction, delayed recall, and orientation. The total score ranges from 0 to 30, with higher scores indicating better cognitive function. The sensitivity and specificity of the Chinese scale were 0.90, 0.84, and the retest reliability coefficient was 0.86.

Physical frailty was defined as an FP score of 1–2 points for pre-frailty and ≥3 points for frailty. Cognitive dysfunction was defined as a MoCA score <26 points for participants with ≥12 years of education, and <25 points for those with <12 years of education. Participants meeting both the frailty (including pre-frailty) and cognitive dysfunction criteria were classified as having cognitive frailty. Participants with a documented diagnosis of dementia or other neurodegenerative diseases were excluded.

#### 2.3.3 Assessment of physical exercise

PARS-3 was originally developed by Hashimoto (41) and later adapted for the Chinese population by Liang (42). The scale assesses physical exercise over the past month based on three indicators: intensity, duration, and frequency. Each indicator is scored on a 5-point scale (intensity: 1–5; frequency: 1–5; duration: 0–4, where 0 indicates no exercise). Higher scores reflect greater intensity, longer duration, or higher frequency, and thus a larger overall exercise volume. The total exercise volume is calculated as: intensity × duration × frequency, yielding a total score ranging from 0 to 100. According to the established criteria, scores ≤19, 20–42, and ≥43 correspond to small, moderate, and large exercise volumes, respectively (43). Its Cronbach’s *α* coefficient was 0.82.

#### 2.3.4 Assessment of self-efficacy

GSES was developed by Schwarzer (44), and the Chinese version revised by Wang Caikang (45) was used in this study. It is a 10-item unidimensional scale with items scored on a 4-point Likert scale (1 = not at all true to 4 = exactly true). Total scores range from 10 to 40, with higher scores indicating stronger self-efficacy (1–10: very low; 10–20: lower; 21–30: higher; 31–40: very high). Its Cronbach’s *α* coefficient is 0.87.

### 2.4 Statistical analysis

SPSS 27.0 software and R software were used for data statistical analysis, the statistical analysis was performed by two-sided test, with *α* = 0.05 as the test level, and *p* < 0.05 as the difference being statistically significant.

**Figure 2:**
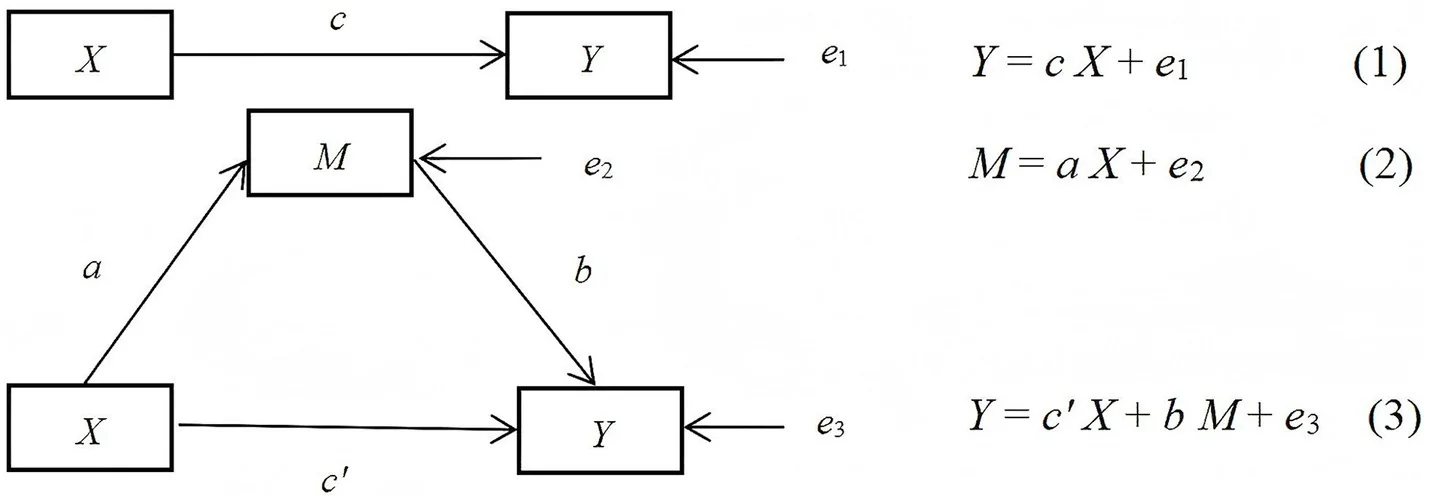
The theoretical diagram of mediation model.

1. Descriptive analysis: The mean (*M*), standard deviation (*SD*), frequency and percentage were used to summarize the characteristics of the participants, including general information, cognitive frailty, physical exercise, and self-efficacy.
2. Univariate analysis: Continuous variables were compared using independent-samples t-tests or Mann–Whitney U tests, depending on the data distribution (normally distributed variables were analyzed using t-tests; non-normally distributed variables were analyzed using Mann–Whitney U tests). Categorical variables were compared using chi-square tests or Fisher’s exact tests, as appropriate.
3. Multivariate analysis: To further examine the factors associated with cognitive frailty, a logistic regression model was constructed with cognitive frailty as the dependent variable, incorporating general information, physical exercise, and self-efficacy as independent variables. Variables with *p* < 0.05 in univariate analyses were identified as candidate predictors. These variables were then entered simultaneously into the multivariable logistic regression model using the Enter method.
4. Analysis of mediation effect: The principle of the mediating variable can be illustrated by the model diagram and corresponding formula shown in Figure 2. The coefficient *c* represents the total effect of the independent variable (*X*) on the dependent variable (*Y*); the coefficient *c’* represents the direct effect of the independent variable (*X*) on the dependent variable (*Y*); the coefficient *a* and *b* represent the effects of the independent variable (*X*) on the mediator variable (*M*) and the mediator variable (*M*) on the dependent variable (*Y*), respectively; the indirect effect is given by the product *a* × *b*, and the total effect is decomposed as *c* = *c’* + *ab*. The mediation effect ratio was calculated as the indirect effect divided by the total effect: Mediation effect ratio = [(*a* × *b*) /(*a* × *b* + *c’*)].

In this study, the independent variable (*X*) and mediator variable (*M*) are continuous variables, and the dependent variable (*Y*) is a binary variable. Therefore, logistic regression analysis should be used instead of linear regression analysis (46).

Mediation analysis is divided into three steps:

The first step was regression analysis: logistic regression analysis of independent variable (*X*) to dependent variable (*Y*) was performed to obtain coefficients *c* and *SE*(*c*). Then the linear regression analysis of the independent variable (*X*) to mediator variable (*M*) was made to obtain the coefficients *a* and *SE*(*a*). Finally, logistic regression analysis of the independent variable (*X*) and mediator variable (*M*) to dependent variable (*Y*) was performed to obtain coefficients *b*, *c’*, *SE*(*b*), *SE*(*c’*).

The second step was to test the mediation effect: we used the distribution-of-the-product method via the RMediation package in R. A mediation effect was considered statistically significant if the 95% CI did not include zero.

The third step was standardization: the regression coefficients obtained from the above models were standardized to achieve comparability across different scales. This was done by multiplying each unstandardized coefficient by the *SD* of the corresponding independent variable and dividing by the *SD* of the dependent variable. The standardized coefficients were then used to estimate the total effect, the direct effect of physical exercise (*X*) on cognitive frailty (*Y*), and the indirect effect via self-efficacy (*M*). The conversion methods of each regression coefficient were as follows:

$$a_{\mathrm{STD}}=a\frac{\mathrm{SD}(X)}{\mathrm{SD}(M)}$$ (4)

$$b_{\mathrm{STD}}=b\frac{\mathrm{SD}(M)}{\mathrm{SD}(Y^{\prime \prime })}$$ (5)

$$c_{\mathrm{STD}}=c\frac{\mathrm{SD}(X)}{\mathrm{SD}(Y^{\prime })}$$ (6)

$$c_{\mathrm{STD}}^{\prime }=c^{\prime }\frac{\mathrm{SD}(X)}{\mathrm{SD}(Y^{\prime \prime })}$$ (7)

In the above formulas, the subscript “STD” denotes standardized coefficients derived from the coefficients in logit units. The standard deviations of the independent variables, *SD*(*X*) and *SD*(*M*), were calculated directly from the raw data. For the dependent variable, the standard deviations *SD*(*Y′*) and *SD*(*Y″*) were estimated using the method proposed by MacKinnon’s method, where π^2^/3 is the variance of the standard logistic distribution (46).

$$\mathrm{Var}(Y^{\prime })=c^{2}\mathrm{Var}(X)+\frac{\pi^{2}}{3}$$ (8)

$$\mathrm{Var}(Y^{\prime \prime })={c^{\prime }}^{2}\mathrm{Var}(X)+b^{2}\mathrm{Var}(M)+2bc^{\prime }\mathrm{cov}(X,M)+\frac{\pi^{2}}{3}$$ (9)

No demographic covariates were included in the mediation models, as the primary aim of this study was to test the statistical significance of the indirect pathway at the bivariate association level, rather than to estimate the effect independent of other variables.

## 3 Result

### 3.1 Descriptive characteristics of participants

The descriptive statistics for the key study variables are presented in Table 1. The physical exercise score was 22.52 ± 7.46. Regarding physical exercise levels, the majority of participants engaged in moderate exercise (60.2%), followed by small exercise (39.1%), with only 0.7% in the large exercise group. The self-efficacy score was 21.32 ± 3.16. For self-efficacy levels, most participants were classified as higher (60.2%) or lower (39.1%). In terms of cognitive frailty, 120 participants (21.3%) met the diagnostic criteria, while 443 (78.7%) did not.

**Table 1:** Descriptive statistics of key study variables (*n* = 563).

Variables	Group	*M* ± *SD*/ *n* (%)
Physical exercise score		22.52 ± 7.46
Physical exercise level	Small exercise	220 (39.1)
	Moderate exercise	339 (60.2)
	Large exercise	4 (0.7)
Self-efficacy score		21.32 ± 3.16
Self-efficacy level	Very low	0 (0.0)
	Lower	220 (39.1)
	Higher	339 (60.2)
	Very high	4 (0.7)
Cognitive frailty	Yes	120 (21.3)
	No	443 (78.7)

M ± SD, mean ± standard deviation.

### 3.2 General characteristics and univariate analysis

Univariate analysis revealed that age, marital status, education level, smoking, drinking, average monthly income, sleep quality, chronic diseases, SCD, physical exercise score, and self-efficacy score were significantly associated with cognitive frailty among community-dwelling older adults (*p* < 0.05). Detailed information is presented in Table 2.

**Table 2:** General characteristics and univariate analysis of factors associated with cognitive frailty in community-dwelling older adults (*n* = 563).

Variables		Non-CF group (*n* = 443)	CF group (*n* = 120)	Statistic	*p*
		n (%)	n (%)		
Age	60–69	318 (92.4)	26 (7.6)	172.580^1^	< 0.001
	70–79	125 (66.8)	62 (33.2)		
	≥80	0 (0.0)	32 (100.0)		
Gender	Male	161 (79.3)	42 (20.7)	0.074^1^	0.786
	Female	282 (78.3)	78 (21.7)		
Marital status	Spouse alive	355 (83.5)	70 (16.5)	24.256^1^	< 0.001
	Single	88 (63.8)	50 (36.2)		
Education level	Primary school or below	95 (65.1)	51 (34.9)	25.565^1^	< 0.001
	Junior high school	185 (80.1)	46 (19.9)		
	Senior high school	112 (87.5)	16 (12.5)		
	Associate degree	38 (86.4)	6 (13.6)		
	Bachelor degree or above	13 (92.9)	1 (7.1)		
Smoking	Yes	124 (70.5)	52 (29.5)	10.343^1^	0.001
	No	319 (82.4)	68 (17.6)		
Drinking	Yes	142 (72.4)	54 (27.6)	6.973^1^	0.008
	No	301 (82.0)	66 (18.0)		
Residence form	Living alone	30 (90.9)	3 (9.1)	3.123^1^	0.077
	Not living alone	413 (77.9)	117 (22.1)		
Average monthly income (¥)	<1,500	37 (48.7)	39 (51.3)	47.288^1^	< 0.001
	1,500 ~ 3,500	319 (83.7)	62 (16.3)		
	>3,500	87 (82.1)	19 (17.9)		
Sleep quality	Well	223 (94.1)	14 (5.9)	59.874^1^	< 0.001
	General	132 (65.0)	71 (35.0)		
	Poor	88 (71.5)	35 (28.5)		
Chronic disease	Yes	212 (65.2)	113 (34.8)	82.986^1^	< 0.001
	No	231 (97.1)	7 (2.9)		
SCD	Yes	106 (53.8)	91 (46.2)	111.834^1^	< 0.001
	No	337 (92.1)	29 (7.9)		
Physical exercise score (*M ± SD*)		23.70 ± 7.26	18.15 ± 6.53	57.321^2^	< 0.001
Self-efficacy score (*M ± SD*)		22.99 ± 2.71	18.86 ± 3.49	110.224^2^	< 0.001

Single, unmarried, widowed, divorced; Non-CF, Non-cognitive frailty; CF, cognitive frailty; M ± SD, mean ± standard deviation.

1 Chi-square test. When the assumptions of the chi-square test were not met, continuity correction chi-square test or Fisher’s exact test was applied. For larger tables with multiple rows and columns, the Fisher–Freeman–Halton exact test was used.

2 Independent samples t-test.

### 3.3 Multivariable logistic regression analysis

Based on the results of univariate analysis, statistically significant related factors were used as independent variables and cognitive frailty as dependent variables. Linear trend tests for the four ordered categorical variables (age, education level, average monthly income and sleep quality) were performed. In the fully adjusted model, the Wald test for linear trend yielded: age (Wald χ^2^ = 75.867, df = 1, *p <* 0.001); education level (Wald χ^2^ = 2.108, df = 1, *p* = 0.147); average monthly income (Wald χ^2^ = 1.674, df = 1, *p =* 0.196) and sleep quality (Wald χ^2^ = 10.388, df = 1, *p* = 0.001). Based on these results, age and sleep quality was retained as a continuous linear term, whereas education level and average monthly income-which violated the linearity assumption-was re-coded as dummy variables in the final model. The highest category of each independent variable was used as the reference group, and variable assignments are presented in Table 3.

**Table 3:** Assignment method of independent variables.

Independent variable	Assignment method
Age	60–69 = 1, 70–79 = 2, ≥80 = 3
Marital status	Single = 1, Spouse alive = 2
Education level	Primary school or below = 1,0,0,0, Junior high school = 0,1,0,0, Senior high school = 0,0,1,0, Associate degree = 0,0,0,1, Bachelor degree or above = 0,0,0,0,
Smoking	No = 0, Yes = 1
Drinking	No = 0, Yes = 1
Average monthly incomes	< 1,500 = 1,0, 1,500 ~ 3,500 = 0,1, > 3,500 = 0,0
Sleep quality	Well = 1, General = 2, Poor = 3
Chronic disease	No = 0, Yes = 1
SCD	No = 0, Yes = 1
Physical exercise score	Entered as continuous
Self-efficacy score	Entered as continuous

SDC, subjective cognitive decline.

Multivariable logistic regression analysis revealed that age (OR = 3.870, 95% CI: 2.052–7.298), chronic diseases(OR = 8.159, 95% CI: 3.276–20.325), SCD (OR = 7.830, 95% CI: 4.115–14.899), physical exercise score (OR = 0.952, 95% CI: 0.908–0.998), and self-efficacy score (OR = 0.812, 95% CI: 0.717–0.921) were significantly associated with cognitive frailty among community-dwelling older adults. As shown in Table 4.

**Table 4:** Multivariate logistic regression analysis of factors associated with cognitive frailty in community-dwelling older adults.

Variables	*β*	*Std.β*	*SE*	*Wald* $\chi^{2}$	*p*	*OR*	95% *CI*
Age	1.353	1.501	0.324	17.482	< 0.001	3.870	2.052–7.298
Chronic disease	2.099	2.099	0.466	20.321	< 0.001	8.159	3.276–20.325
SCD	2.058	2.058	0.328	39.306	< 0.001	7.830	4.115–14.899
Physical exercise score	−0.049	−0.177	0.024	4.129	0.042	0.952	0.908–0.998
Self-efficacy score	−0.208	−0.570	0.064	10.568	0.001	0.812	0.717–0.921
constant	−1.641	−5.506	2.654	0.382	0.536	0.194	

β, regression coefficient; Std. β, Standardized regression coefficient; SE, standard error; Wald $\chi^{2}$ , Wald chi-square statistic; *p*, *p*-value; OR, odds ratio; 95% CI, 95% confidence interval.

### 3.4 Mediation analysis

In order to further verify the relationship among physical exercise, self-efficacy and cognitive frailty, SPSS 27.0 software and the RMediation software package of R software were used to conduct mediation analysis with self-efficacy as the mediator variable (*M*), physical exercise as the independent variable (*X*) and cognitive frailty as the dependent variable (*Y*).

In the first step, logistic regression analysis of physical exercise (*X*) to cognitive frailty (*Y*) was performed, and *c* = −0.112 and *SE*(*c*) = 0.016 were obtained. Then the linear regression analysis of physical exercise (*X*) to self-efficacy (*M*) was made, and the coefficient *a* = 0.126, *SE*(*a*) = 0.017 were obtained. Finally, logistic regression analysis of physical exercise (*X*) and self-efficacy (*M*) to cognitive frailty (*Y*) was conducted, and the coefficients were *b* = −0.340, *c’* = −0.079, *SE*(*b*) = 0.047, *SE*(*c’*) = 0.017.

In the second step, the total effect (*p* < 0.001) and direct effect (*p* < 0.001) of physical exercise on cognitive frailty were statistically significant. The 95%*CI* obtained using the distribution-of-the-product method via the RMediation package in R was [−0.059, −0.029], which did not include zero, confirming that self-efficacy significantly mediated the association between physical exercise and cognitive frailty.

**Figure 3:**
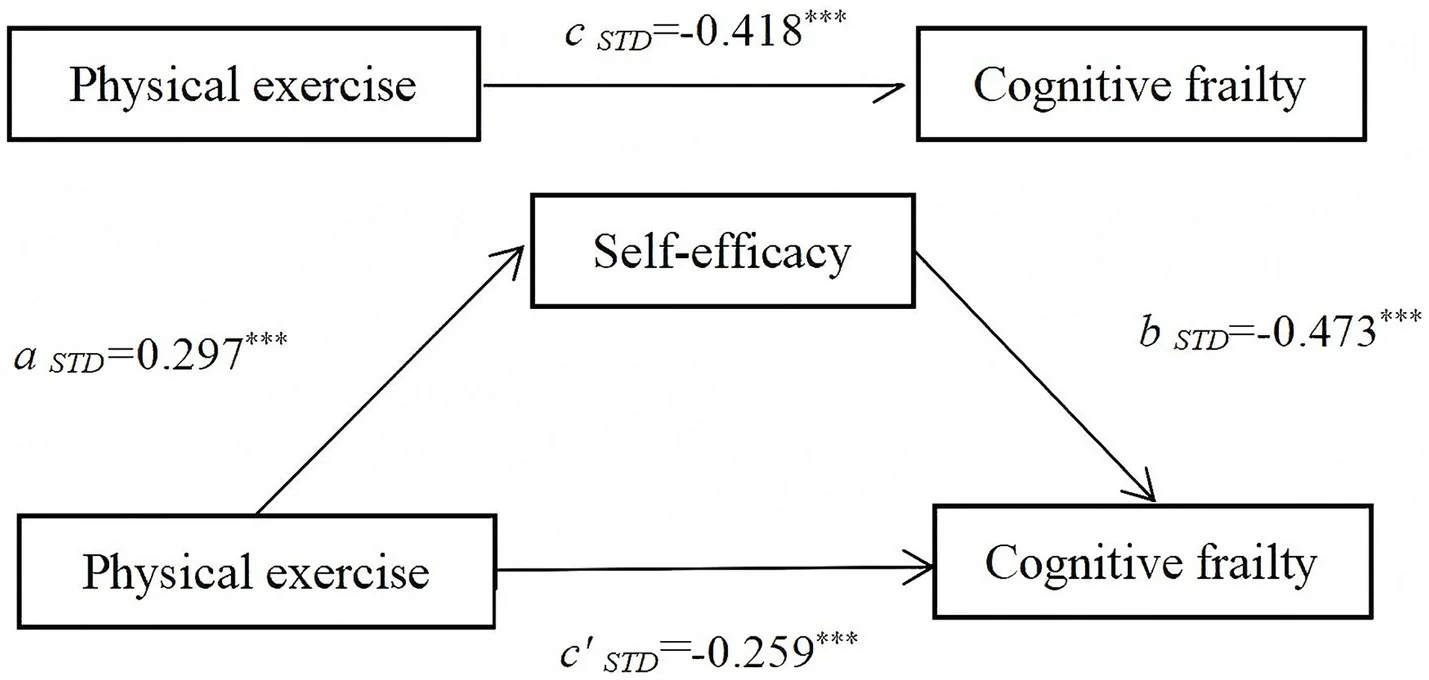
The mediation analysis path. *p < 0.05; **p < 0.01; ***p < 0.001.

In the third step, according to the calculation of SPSS software, *SD* (*X*) = 7.460; *SD* (*M*) = 3.164; Var (*X*) = 55.651; *Var* (*M*) = 10.011; *Cov* (*X*, *M*) = 7.031. With Equations 8, 9 to calculate the *Var* (*Y′*) = 3.985, *Var* (*Y″*) = 5.160, then *SD* (*Y′*) = 1.996, *SD*(*Y″*) = 2.272. According to Equations 4–7, *a _STD_* = 0.297; *b _STD_* = −0.473; *c’*_STD_ = −0.259; *c _STD_* = −0.418. The mediation analysis path is shown in Figure 3.

Physical exercise was positively associated with self-efficacy (*a*_STD_ = 0.297, *p* < 0.001), and self-efficacy was negatively associated with cognitive frailty (*b*_STD_ = −0.473, *p* < 0.001). The direct effect of physical exercise on cognitive frailty, after adjusting for self-efficacy, remained significant (*c’*_STD_ = −0.259, *p* < 0.001). The indirect effect (*a* × *b*) was −0.140 (95%*CI*: −0.059, −0.029). The total effect of physical exercise on cognitive frailty was *c*_STD_ = −0.418. The mediation effect ratio was 35.17% [(*a _STD_* ×*b*_STD_) /(*a _STD_* ×*b _STD_*+*c’ _STD_*)], suggesting that approximately 35.17% of the total association between physical exercise and cognitive frailty was mediated through self-efficacy.

## 4 Discussion

Cognitive frailty is a novel indicator of physical health in older adults, and its effective prevention and delay represent an important goal in healthy aging. This study found that physical exercise was associated with lower odds of cognitive frailty, and that self-efficacy partially mediated this association among community-dwelling older adults. This finding extends the existing literature by clarifying the psychological pathway through which physical activity may be linked to cognitive health, and provides an empirical basis for community-based interventions targeting both exercise promotion and self-efficacy enhancement.

### 4.1 Status of physical exercise and cognitive frailty among community-dwelling older adults

The PARS-3 classification revealed a highly skewed distribution, with only 0.7% of participants classified as large-exercise. This pattern is consistent with previous observations in community-dwelling older Chinese populations, who tend to engage predominantly in light-to-moderate physical activity (47). Importantly, because exercise volume was treated as a continuous variable in all regression and mediation analyses, this unbalanced categorical distribution did not directly affect the stability of the main statistical estimates. The continuous treatment preserves the full dose–response information and avoids the statistical instability that would arise from sparse categories in regression models. Nonetheless, the limited representation of high-level exercise in the sample reflects the restricted range of exercise volume in this population, which may reduce statistical power to detect potential intensity-specific effects.

The prevalence of cognitive frailty in our sample (21.3%) was comparable to the pooled estimate reported in a recent meta-analysis of Chinese older adults (21, 95% CI: 19–23) (48). However, it was slightly lower than the 26% pooled prevalence reported in another Chinese population meta-analysis (49). In contrast, our prevalence was substantially higher than the global community-based estimate of 5% (95% CI: 4–6) reported in a large-scale meta-analysis covering 17 countries. Notably, the same global meta-analysis reported a pooled prevalence of 17% (95% CI: 13–21) for potentially reversible cognitive frailty and 21% (95% CI: 15–29) for reversible cognitive frailty in community settings (50), suggesting that a substantial proportion of cognitive frailty cases may be modifiable through early intervention. Several factors may account for the variations in cognitive frailty prevalence across studies. First, the use of different assessment tools and diagnostic criteria may have contributed to the observed discrepancies. For frailty, various instruments have been employed, including the FP, the Comprehensive Frailty Assessment Instrument Plus (CFAI-Plus), and the Clinical Frailty Scale (CFS); for cognitive impairment, the MoCA and the Mini-Mental State Examination (MMSE) are commonly used. The combination of different frailty and cognitive measures to define cognitive frailty inevitably results in heterogeneous diagnostic criteria. Second, differences in sample characteristics-such as age distribution, cultural background, socioeconomic status, geographic region, and ethnicity-may also influence the occurrence of cognitive frailty among community-dwelling older adults.

Cognitive frailty represents a potential target for the secondary prevention of dementia, particularly its reversible sub-type. Community healthcare workers can identify the underlying causes through comprehensive assessment and implement evidence-based, individualized intervention models to delay the progression of cognitive frailty. Such approaches may help reduce adverse health outcomes and improve the quality of life among community-dwelling older adults.

### 4.2 Factors associated with cognitive frailty in community-dwelling older adults

Univariate analysis revealed that age, marital status, education level, smoking, drinking, average monthly income, sleep quality, chronic diseases, SCD, physical exercise score, and self-efficacy score were significantly associated with cognitive frailty among community-dwelling older adults. In the multivariable model, age, sleep quality, chronic diseases, SCD, physical exercise score, and self-efficacy score remained independently associated with cognitive frailty.

Cognitive frailty may represent a state of age-related decline in brain neurophysiological reserves, encompassing features of both neurodegenerative and vascular pathologies. Several studies have suggested that age-related neuropathologies may exert adverse effects on both physical frailty and cognitive decline (51, 52). Sleep disorders are associated with elevated inflammatory factors, and pro-inflammatory cytokines may contribute to frailty through enhanced protein hydrolysis. In addition, sleep disorders may lead to reduced neurotransmitter levels, cerebrovascular sclerosis, and cerebral hypoxia, which can impair memory and cognitive function, potentially contributing to the development of cognitive frailty in older adults (53). Studies have shown that older adults with chronic diseases have a 1.5-fold higher risk of mild cognitive impairment compared with those without chronic conditions (54). Moreover, chronic diseases themselves may lead to functional decline across multiple physiological systems, impair daily living activities, and contribute to adverse health outcomes such as frailty. SCD refers to a self-reported decline in cognitive function that does not reach the threshold for MCI or dementia on objective assessments. According to the cognitive frailty definition, older adults with SCD are classified as having cognitive frailty once they concurrently present with frailty or pre-frailty. This may explain why older adults with SCD are at higher risk of developing cognitive frailty.

Regular physical exercise of adequate intensity and duration is a protective factor associated with lower odds of cognitive frailty among community-dwelling older adults. Ji et al. (55) reported that regular exercise interventions lasting more than 8 weeks may improve global cognitive function and physical frailty status in older adults. A systematic review and meta-analysis by Yuan et al. (56) demonstrated that exercise interventions significantly improved overall cognition, physical frailty, walking ability, and gait speed among older adults with cognitive frailty. Similarly, Wu et al. (57) found that resistance training significantly enhanced global cognitive function and gait speed in this population. These findings are consistent with our observation that physical exercise is associated with lower odds of cognitive frailty. This may be because long-term adherence to physical exercise enhances cardiorespiratory fitness and psychological resilience, while also improving muscle strength and core stability, thereby delaying the onset and progression of physical frailty in older adults (58). In addition, physical exercise may improve cardiovascular function in older adults, increasing cerebral blood flow velocity and oxygen supply, thereby providing more nutrients to brain tissue and supporting the maintenance of cognitive function. Furthermore, long-term regular exercise has been shown to modulate memory-related factors such as hippocampal structure, enhance cerebral metabolism, and regulate the levels of various protein-like molecules in the brain, ultimately contributing to improved cognitive performance (26, 59, 60).

This study found a significant difference in self-efficacy between community-dwelling older adults with and without cognitive frailty, with higher self-efficacy levels being associated with lower odds of cognitive frailty. Which is consistent with previous research results (35, 61). This may be because individuals with lower self-efficacy exhibit greater fluctuations in biochemical secretion when facing stressors, triggering immune system dysregulation and increasing disease susceptibility. Conversely, higher self-efficacy is associated with more stable biochemical profiles, which may help maintain immune function, preserve physical health, and reduce the likelihood of frailty (36). In addition, self-efficacy may influence cognitive function through its combined effects on motivation and information processing. Higher self-efficacy is associated with stronger health self-management awareness and more positive cognitive appraisals when facing stressful events, potentially contributing to the delay of cognitive decline (62).

In conclusion, cognitive frailty is shaped by a complex interplay of multiple factors. Effective prevention and intervention efforts should adopt a holistic approach that addresses these determinants, thereby reducing the risk of cognitive frailty and its associated adverse outcomes in older adults.

### 4.3 Analysis of mediation effect

The mediation analysis revealed that self-efficacy partially mediated the association between physical exercise and cognitive frailty, with an indirect effect accounting for 35.17% of the total effect. This finding can be interpreted within the framework of Bandura‘s triadic reciprocal determinism, which posits that behavior, personal factors, and the environment are mutually influential. In this study, physical exercise (behavior), self-efficacy (personal factor), and cognitive frailty (an environmental/contextual health outcome) formed an interactive triad.

The pathway from physical exercise to self-efficacy aligns with the behavioral-personal dimension of the triad. Engaging in regular physical exercise provides older adults with direct mastery experiences-such as successfully completing a walking program or adhering to an exercise routine-which are among the most potent sources of self-efficacy. Bandura identified enactive mastery experience as the most influential source of efficacy beliefs, as it provides authentic evidence of one’s capabilities (31). Moreover, the social and physiological feedback from exercise (e.g., improved physical function, positive affective states, peer encouragement) further reinforces individuals’ confidence in their ability to maintain health-promoting behaviors (63, 64).

The pathway from self-efficacy to cognitive frailty reflects the personal–environmental dimension of the triad. Individuals with higher self-efficacy are more likely to adopt and sustain health-promoting behaviors (e.g., regular exercise, cognitive engagement, social participation), which directly benefit cognitive and physical health (65). This is consistent with the tenet that personal factors shape behavior, which in turn modifies environmental conditions. This mechanism is supported by recent empirical evidence. Fotokian et al. (66), in a multi-center cross-sectional study of Iranian older adults, reported that healthier lifestyle scores were positively associated with greater motivation for physical activity, reinforcing the role of psychological resources-such as self-efficacy-in promoting sustained health engagement.

The direct pathway from physical exercise to cognitive frailty (independent of self-efficacy) represents the behavioral-environmental link in the triadic framework. According to this perspective, behavior not only is shaped by personal and environmental factors but also actively modifies the individual’s internal physiological environment. Physiologically, regular exercise improves cardiovascular function, enhances cerebral blood flow, reduces systemic inflammation, and promotes neuroplasticity-including increased hippocampal volume and enhanced synaptic plasticity (67). These adaptations counteract age-related neurodegeneration and physical decline, thereby delaying cognitive frailty. Exercise also directly improves physical function (gait speed, muscle strength, and balance) which are core components of the FP (55). By mitigating physical frailty, exercise indirectly preserves cognitive function, as physical and cognitive decline often share common pathophysiological pathways. This dual effect underscores exercise as a direct behavioral intervention for cognitive frailty.

Based on our findings, we propose the following practice recommendations for community geriatric care: (1) Tiered exercise interventions by PARS-3 subgroups. For older adults with “small exercise levels” (PARS-3 ≤ 19), we recommend a stepwise approach that prioritizes the establishment of exercise frequency before increasing intensity or duration. The initial focus should be on achieving 3–5 sessions per week at a light-to-moderate intensity (e.g., walking at a conversational pace), with session duration starting at 10–15 min and gradually progressing to 20–30 min over 4–6 weeks. This progressive strategy minimizes injury risk and dropout, while allowing individuals to build exercise self-efficacy through attainable short-term goals. Regular follow-up by community health workers is recommended to monitor adherence and adjust the plan as needed. For those with” moderate exercise levels” (PARS-3 20–42), the focus should be on maintenance and diversification of exercise types. For “large exercise levels” (PARS-3 ≥ 43), injury prevention and sustainable engagement should be prioritized, with encouragement for them to serve as peer mentors (2). Community-based strategies to enhance self-efficacy. Given the mediating role of self-efficacy, we recommend three feasible strategies: “peer support groups” led by trained older adult volunteers to provide modeling and emotional encouragement; “phased health goal incentives” (e.g., weekly to quarterly milestones with small rewards) to build mastery experiences; and “regular health education lectures” covering exercise-cognition benefits and practical tips for overcoming exercise barriers.

### 4.4 Limitations

There are some limitations to be mentioned. First, this study employed a cross-sectional design, which precludes establishing causal relationships between physical exercise and cognitive frailty. Moreover, the lack of longitudinal follow-up prevented us from tracking changes in cognitive frailty status over time. Cognitive frailty is a dynamic condition with potentially reversible or fluctuating trajectories, and our single time-point measurement could not capture this temporal variability. Future research should incorporate longitudinal designs with repeated assessments to further clarify the causal mechanisms underlying the physical activity–cognitive frailty relationship and to provide evidence-based guidance for developing personalized intervention strategies.

Second, due to human, financial, and time constraints, this study employed a convenience sampling method, recruiting older adults from nine communities in Kaifeng City, Henan Province. This non-probability approach limits the generalizability of our findings to the broader older adult population. Furthermore, the relatively small proportion of rural participants in our sample further restricts the applicability of our results to rural older adult populations, who face distinct challenges in healthcare access and physical activity resources compared with their urban counterparts. Accordingly, the conclusions of this study should be interpreted as specific to the local community-dwelling older adults surveyed in Henan Province, rather than generalized to all community-dwelling older adults nationwide. Future research employing probability sampling, multi-regional recruitment, and larger sample sizes is warranted to enhance the representativeness and generalizability of the findings.

Third, demographic variables (e.g., age, marital status) were not controlled for in the mediation model. Although our primary analysis focused on testing the indirect pathway at the bivariate level, the absence of these covariates may have introduced residual confounding. Future studies should incorporate relevant demographic covariates into mediation analyses to obtain more comprehensive estimates.

Fourth, this study relied on self-reported scales without objective physiological or biomarker testing, which may introduce recall and social desirability biases. Future studies should incorporate objective physical activity monitors (e.g., accelerometers) to provide precise quantification of exercise volume and intensity, and include blood-based biomarkers (e.g., inflammatory markers such as CRP and IL-6, and neurotrophic factors such as BDNF) to elucidate the biological pathways linking physical exercise to cognitive frailty.

Finally, SCD was assessed using a single self-report item rather than a validated multi-domain SCD scale. Although this single-item approach has been used in community-based epidemiological studies as a practical screening tool, it may not fully capture the multidimensional nature of SCD, including its severity, frequency, and the specific cognitive domains affected. Future studies employing validated SCD scales are warranted to provide a more comprehensive assessment.

## 5 Conclusion

This study found that cognitive frailty was prevalent among community-dwelling older adults. Both physical exercise and self-efficacy were significantly associated with cognitive frailty, with higher levels of each being linked to a lower likelihood of the condition. Mediation analysis further revealed that self-efficacy partially mediated the association between physical exercise and cognitive frailty, suggesting that physical exercise may influence cognitive frailty through both direct and indirect pathways via self-efficacy.

## Data Availability

The original contributions presented in the study are included in the article/supplementary material, further inquiries can be directed to the corresponding author.
